# 

**Published:** 1887-05-28

**Authors:** 


					> }' ?
No. 35, Vol. II.
PRICE ONE PENNY. [)
lyf 1?^ ff: ??!l.
tft?.
May 28th 1887.
% JL'JL

				

